# The effect of abductor muscle and anterior-posterior hip contact load simulation on the in-vitro primary stability of a cementless hip stem

**DOI:** 10.1186/1749-799X-5-40

**Published:** 2010-06-24

**Authors:** Youngbae Park, Carolyne Albert, Yong-San Yoon, Göran Fernlund, Hanspeter Frei, Thomas R Oxland

**Affiliations:** 1 Department of Mechanical Engineering, Korean Advanced Institute of Science and Technology, Daejeon, Republic of Korea; 2 Orthopaedic and Rehabilitation Engineering Center, Marquette University, Milwaukee, Wisconsin, USA; 3 Department of Materials Engineering, University of British Columbia, Vancouver, Canada; 4 Department of Mechanical and Aerospace Engineering, Carleton University, Ottawa, Canada; 5 Department of Mechanical Engineering, University of British Columbia, Vancouver, Canada

## Abstract

**Background:**

In-vitro mechanical tests are commonly performed to assess pre-clinically the effect of implant design on the stability of hip endoprostheses. There is no standard protocol for these tests, and the forces applied vary between studies. This study examines the effect of the abductor force with and without application of the anterior-posterior hip contact force in the in-vitro assessment of cementless hip implant stability.

**Methods:**

Cementless stems (VerSys Fiber Metal) were implanted in twelve composite femurs which were divided into two groups: group 1 (N = 6) was loaded with the hip contact force only, whereas group 2 (N = 6) was additionally subjected to an abductor force. Both groups were subjected to the same cranial-caudal hip contact force component, 2.3 times body weight (BW) and each specimen was subjected to three levels of anterior-posterior hip contact load: 0, -0.1 to 0.3 BW (walking), and -0.1 to 0.6 BW (stair climbing). The implant migration and micromotion relative to the femur was measured using a custom-built system comprised of 6 LVDT sensors.

**Results:**

Substantially higher implant motion was observed when the anterior-posterior force was 0.6BW compared to the lower anterior-posterior load levels, particularly distally and in retroversion. The abductor load had little effect on implant motion when simulating walking, but resulted in significantly less motion than the hip contact force alone when simulating stair climbing.

**Conclusions:**

The anterior-posterior component of the hip contact load has a significant effect on the axial motion of the stem relative to the bone. Inclusion of the abductor force had a stabilizing effect on the implant motion when simulating stair climbing.

## Background

Loosening of femoral hip implants is a major problem in total hip arthroplasty [1]. Clinical studies have shown that early implant migration negatively affects the long term performance of cementless femoral stems [2-4]. Excessive micromotion at the bone-implant interface inhibits successful bone ingrowth in cementless implants and may therefore result in early implant loosening [5-7]. The immediate post operative migration and micromotion (primary stability) of different femoral stems have been evaluated under simulated physiological loading in *in-vitro *experiments [8-12]. Although it has not yet been demonstrated for cementless stems, some cemented stems with inferior clinical results have been shown to also result in higher *in-vitro *micromotions [13], which demonstrates the clinical relevance of these *in-vitro *tests.

The physiological loads acting on the head of a femoral stem have been established by telemetric measurements for daily activities such as walking and stair climbing [14-17], while the muscle forces for these activities have been estimated by numerical models [15,18-20]. It is challenging to include all hip contact and muscle forces acting on the femur in an in-vitro test and simplified test setups have therefore been used to simulate the biomechanical environment to which hip implants are subjected to post-operatively. Some *in-vitro *studies have simulated the hip contact force alone [9,11,12,21-23], while others included one [24,25] or many muscle forces [8,10]. However, it is not clear how these variations affect stem migration and micromotion.

In particular, the precise effect of the abductor muscle load (F_abd_) on the primary stability of uncemented stems has not been demonstrated. Of all muscle groups, the abductors have been shown to have the most pronounced effect on femoral strains, increasing medial bending in the proximal femur during gait [26-28]. There are, however, contradictory results concerning the effects of including muscle loading on primary stability, and these studies also incorporated more than one muscle group such that the effect of the abductor muscles has not been isolated. In an *in-vitro *study of cemented stems, the simulation of muscle forces (abductor, vastus lateralis, and tensor fascia latae) resulted in a small and non-significant *reduction *in migration compared with the hip contact force applied alone [8]. On the other hand, in another *in-vitro *study, the inclusion of muscle loads (abductor, tensor fascia latae, ilio-tibial tract, vastus lateralis and vastus medialis) *increased *migration and micromotion of a cementless stem [10]. We hypothesise that simulation of an abductor muscle force increases implant micromotion and migration of cementless stems compared with hip contact forces alone.

The effect of the anterior-posterior component of the hip contact force (F_ap_) on implant primary stability has also not been established definitely. *In-vitro *studies have measured the torsional strength of cementless implant fixation [29-31] and these values were found to approach the torque levels measured in-vivo during stair climbing [32] Physiological cranial-caudal loads, however, were not applied in these *in-vitro *studies, which may underestimate the torsional strength of the stem-femur constructs. Studies have measured implant migration and micromotion under varying F_ap _loads [10,24]. One study reported higher distal migration and micromotion when simulating stair climbing compared to walking loads [10], whereas the other did not observe a difference in distal micromotion between stair climbing and single-leg stance, a configuration without F_ap _[24]. These studies, however, also varied muscular loading such that the effect of F_ap _was not isolated. We hypothesise that the higher F_ap _load observed during stair climbing generates greater implant-bone micromotion and migration compared with walking.

To test our hypotheses, we conducted *in-vitro *tests on composite femurs, in which we examined the effect of the abductor on the motion of a cementless implant at three levels of anterior-posterior hip contact load.

## Methods

A cementless femoral stem (VerSys collarless size 14, Zimmer Co., Dover, Ohio, USA) was implanted in twelve composite femurs (Model 3303, Third Generation, Pacific Research Laboratories, Vashon, Washington, USA). The femoral cavity was prepared manually according to the implant manufacturer's instructions, using straight reamers and broaches. Visual inspection of the cavity after preparation revealed that the regions of contact between the stem and the cortical component of the composite bones were consistent between specimens. The specimens were cut at 27 cm from the proximal end and the distal 6 cm were potted in dental stone (Tru-Stone, Heraeus Kulzer, Armonk, New York). The specimens were then loaded cyclically on a biaxial servohydraulic testing machine (Instron Model 8874, Instron, Canton, Massachusetts). The loads applied were designed to mimic walking and stair climbing loads as measured by Bergmann et al. [15].

**Figure 1:**
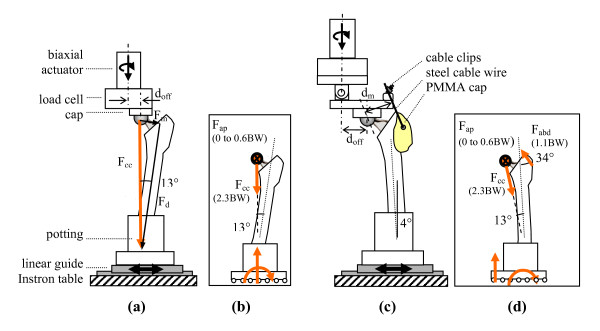
**Loading set-ups**. (a) Group 1 - no abductor, i.e. hip contact force alone. Axial and torsional loading of the actuator produced distal (F_d_), medial (F_m_) and anterior-posterior (F_ap_) loading of the femoral head due to the mounting geometry and the offset between the femoral head and the central axis of the actuator, d_off _(32 mm). (b) Resulting forces on the femur for group 1. (c) Group 2 - hip contact force and abductor. (d) Resulting forces on the femur for group 2 (equilibrium calculations are presented in Figure 2).

**Figure 2:**
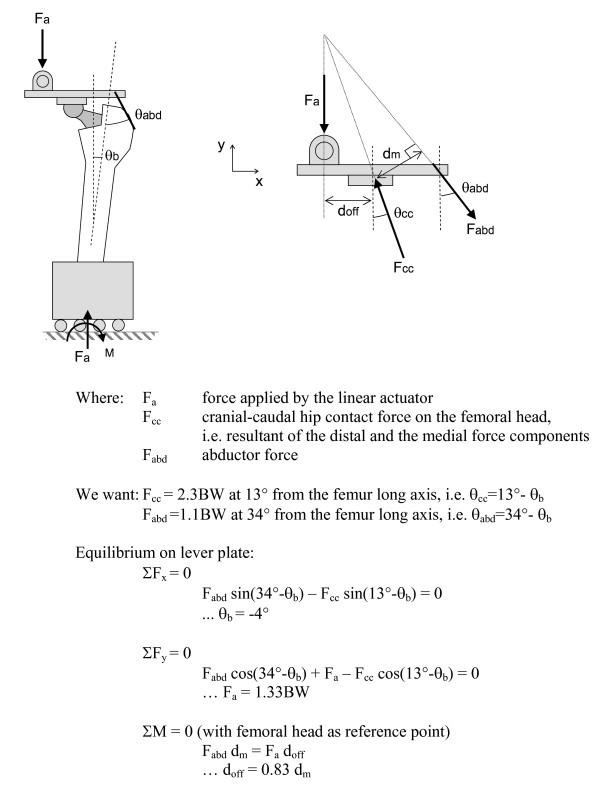
**Equilibrium calculations for group 2 (abductor)**.

The specimens were divided into two groups for biomechanical testing. Group 1 (N = 6, Figure 1a-b) was loaded with the hip contact force only. A cranial-caudal force (F_cc_) of 2.3 times body weight (BW) was applied by the linear actuator, with the femur potted at 13° of adduction (Figure 1a), generating a proximal-distal component of 2.2 BW and a medial-lateral component of 0.5 BW. A body weight of 75 kg was used for the simulations. The potted distal femur was fastened to a linear guide to avoid a horizontal reaction force in the frontal plane. Group 2 (N = 6, Figure 1c-d) was additionally loaded with an abductor muscle load (F_abd_). The F_abd _was applied with a steel cable using a lever that was joined to the actuator through a hinge (Figure 1c). The steel cable was attached to the greater trochanter through a custom-moulded polymethylmethacrylate (PMMA) cap. The cable passed through a copper tube that was embedded into the PMMA cap, and the cap was attached to the bone with a 4 mm diameter steel pin inserted anterior-posteriorly through the greater trochanter. The same muscle attachment cap was used for all specimens to obtain a repeatable muscle orientation relative to the femur. An F_abd _of 1.1 BW [20] was applied by adjusting the offset between the actuator and the femoral head, d_off_, in proportion to the muscle-to-femoral head lever arm, d_m_, see Figure 1c. The measured d_m _varied between 46 and 50 mm, and d_off _was adjusted in proportion to d_m _to maintain the same F_cc _and F_abd _values between specimens. Based on equilibrium calculations (shown in Figure 2), the same F_cc _orientation as group 1 was achieved for group 2 by potting the femurs at 4° of abduction.

For both groups, the anterior-posterior hip contact load (F_ap_) was applied by the rotary actuator (M = F_ap_*d_off_). For group 1 d_off _was 32 mm, whereas in group 2 it was set at 0.83*d_m_, (and since d_m _ranged between 46 and 49 mm, d_off _therefore ranged between 38 and 41 mm). The F_ap _was applied in three phases of 1000 cycles each. The first load phase simulated walking without F_ap _(F_ap _= 0), the second simulated walking with F_ap _(F_ap _= -0.1 to 0.3 BW), and the third simulated stair climbing (F_ap _= -0.1 to 0.6 BW). These peak F_ap _loads are based on published results of *in-vivo *measurements [15]. During stair climbing, an actuator rotation of approximately 1° in amplitude was observed in the muscle group. Based on the geometry of the implant and loading set-up, we estimate that this rotation would have affected the orientation of the abductor load relative to the femur by approximately 1°.

The applied peak loads for both groups are summarized in Table 1. The loads were sinusoidal with a frequency of 1 Hz with in-phase peak loads.

**Table 1:** Loads applied to the hip system

	Loading step	Cycles	Hip contact force (xBW)		**F**_**abd**_
				
			**F**_**cc**_	**F**_**ap**_**(xBW)**	
**Group 1 No abductor**	**1**	**1-1000**	**0.4 to 2.3**	**0**	**n/a**
	**2**	**1001-2000**	**0.4 to 2.3**	**-0.1 to +0.3**	**n/a**
	**3**	**2001-3000**	**0.4 to 2.3**	**-0.1 to +0.6**	**n/a**

**Group 2 Abductor**	**1**	**1-1000**	**0.4 to 2.3**	**0**	**1.1**
	**2**	**1001-2000**	**0.4 to 2.3**	**-0.1 to +0.3**	**1.1**
	**3**	**2001-3000**	**0.4 to 2.3**	**-0.1 to +0.6**	**1.1**

F_cc _is in the caudal direction, and a positive F_ap _is in the posterior direction. The peak loads (maximum and minimum) were defined based on published data [15], and scaled for a 70 kg individual.

**Figure 3:**
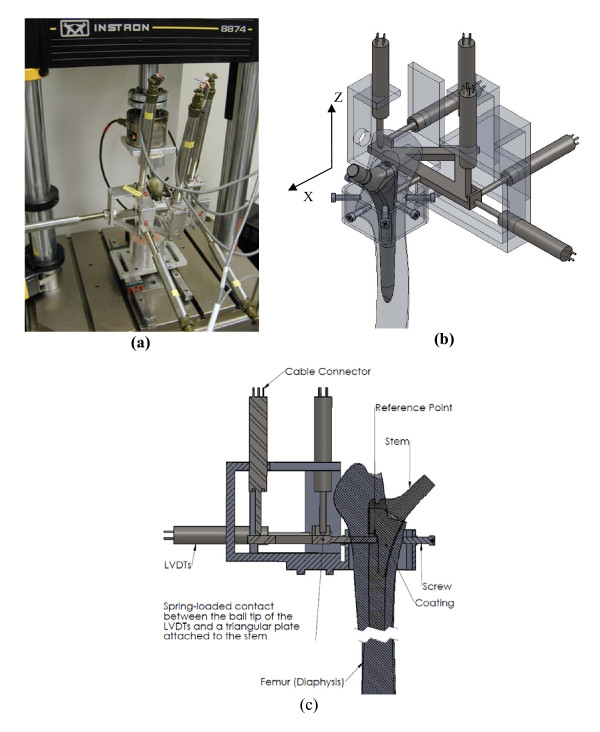
**Motion measurement set-up**. (a) LVDT set-up. (b) Coordinate system and sensor diagram. The reference point is located on the lateral side of the stem, 113 mm proximal from the stem tip. The arrows show the location and direction of each sensor.

The relative motion between stem and bone was measured with a custom-built system similar to previously published designs [33-35]. The system, illustrated in Figure 3, was comprised of six linear variable differential transformers (LVDTs) mounted on a frame that was rigidly attached to the femur with seven set screws. The sensors measured the three dimensional motion of a triangular plate that was rigidly attached to the lateral surface of the implant through a hole in the cortex. The implant motion was calculated from the motion of the triangle using a custom program implemented in Matlab (MathWorks, Natick, Massachussetts). The measurement resolution was smaller than 0.7 μm in all translational directions, and smaller than 0.001° in rotation. The accuracy of the system in measuring translation was evaluated against a micrometer precision dial gauge (Kafer, Germany). Translation along each of the three axes was applied to the implant, with the sensors attached to an over-reamed composite femur. The maximum translation error observed was 2 μm over a range of 30 μm (mean 0.8 μm, stdev 0.8 μm for 9 measurements), and 10 μm over a range of 300 μm (mean 5.6 μm, stdev 3.0 μm for 9 measurements). The accuracy of each sensor was also measured with a dial gauge (Kafer, Germany), where a maximum error of 1.7 μm was observed over a range of 200 μm (mean 0.6 μm, stdev 0.4 μm for 60 measurements). The rotation accuracy was evaluated analytically from the maximum individual LVDT errors, yielding a maximum rotation error of 0.0026°.

**Figure 4:**
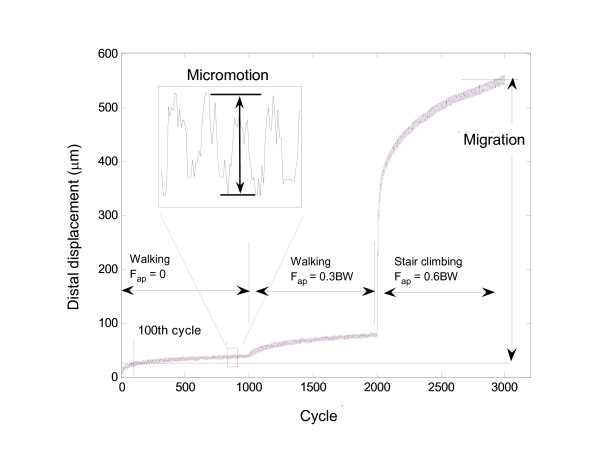
**Distal movement of the stem relative to the bone**. Micromotion was calculated as the average amplitude of the cyclic motion during the last 200 cycles of each loading step (F_ap _= 0, F_ap _= 0.3 BW, and F_ap _= 0.6 BW). Migration was the cumulative stem displacement at the end of each step, with respect to its position at cycle 100.

Migration was defined as the difference in stem mean position (translations and rotations) between cycle 100 and the last cycle of each loading step, i.e. cycle 1000 (F_ap _= 0), cycle 2000 (F_ap _= 0.3 BW) and cycle 3000 (F_ap _= 0.6 BW), see Figure 4. The first 100 cycles were used for pre-conditioning [10,12]. Micromotion was defined as the average reversible motion of the stem during the last 200 cycles of each loading step, i.e. cycles 800-1000, 1800-2000, and 2800-3000 (Figure 4). The migration and micromotion were each comprised of 6 components: translation along the medial, anterior and distal axes (at the reference point shown in Figure 3), as well as rotations projected in the frontal, sagittal and transverse planes. The resultants of the three translational migration and micromotion components are presented as 'total translational migration' and 'total translational micromotion'. Similarly, the terms 'total rotational migration' and 'total rotational micromotion' were used to represent the resultant of all rotational components, and were defined as the rotations about the helical axis [36].

The effects of F_abd _and F_ap _on each migration and micromotion component and their resultants were examined with a two-way ANOVA, with F_ap _as a repeated measure, followed by Student Newman Keuls post hoc analysis with a significance level of 95%.

## Results

**Figure 5:**
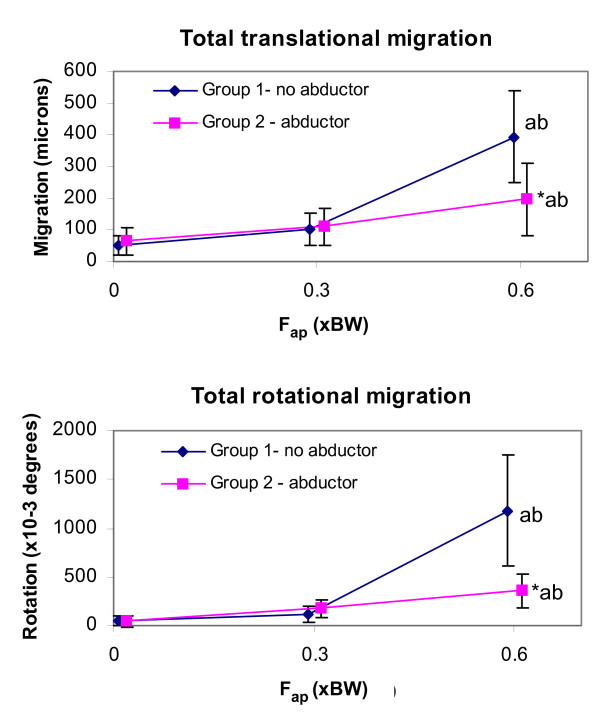
**Implant migration resultants as a function of F**_**ap **_**for each group**. (top) Total translational migration, i.e. (medial^2 ^+ anterior^2 ^+ distal^2^)^1/2^. (bottom) Total rotational migration (about the helical axis). Results shown are means (N = 6) and 95% confidence intervals. * p < 0.05 compared to the other group at the same F_ap _value. ^a ^p < 0.05 compared to the same group at F_ap _= 0 BW. ^b ^p < 0.05 compared to the same group at F_ap _= 0.3 BW.

**Figure 6:**
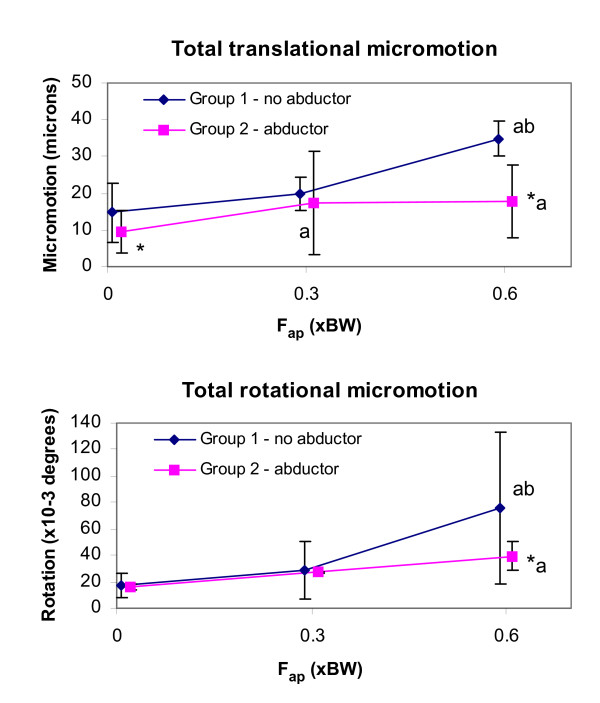
**Implant micromotion resultants as a function of F**_**ap **_**for each group. **(a) Total translational micromotion (b) Total rotational micromotion (about the helical axis). Results shown are means (N = 6) and 95% confidence intervals. * p < 0.05 compared to the other group at the same F_ap _value. ^a ^p < 0.05 compared to the same group at F_ap _= 0 BW. ^b ^p < 0.05 compared to the same group at F_ap _= 0.3 BW.

The implant-bone migration and micromotion components for both groups at all loading conditions are summarized in Tables 2 and 3. The resultants of these components, i.e. total translational and rotational migrations and micromotions, are presented in Figures 5 and 6.

**Table 2:** Migration results

		Group 1 No abductor		Group 2 Abductor	
**Component**	**F_ap_**	**Average**	**95% CI**	**Average**	**95% CI**

**Lateral translation (μm)**	**0**	**7**	**± 4**	**5**	**± 8**
	0.3 BW	17	± 10	9	± 13
	0.6 BW	62 ^ab^	± 18	20* ^a^	± 14

Anterior translation (μm)	0	-2	± 7	0	± 2
	0.3 BW	7	± 7	10	± 20
	0.6 BW	39 ^ab^	± 26	19 *	± 16

Distal translation (μm)	0	50	± 28	63	± 43
	0.3 BW	100	± 52	103	± 67
	0.6 BW	385 ^ab^	± 147	191* ^ab^	± 123

Sagittal plane rotation (×10^-3^°)	0	12	± 7	5	± 5
	0.3 BW	30	± 13	14*	± 7
	0.6 BW	-8 ^ab^	± 24	32* ^ab^	± 22

Frontal plane rotation (×10^-3^°)	0	15	± 6	-2*	± 5
	0.3 BW	34	± 16	-10*	± 16
	0.6 BW	25	± 56	-14*	± 19

Transverse plane rotation (×10^-3^°)	0	34	± 53	43	± 55
	0.3 BW	-19	± 144	175	± 89
	0.6 BW	-1175 ^ab^	± 567	359*	± 172

* p < 0.05 compared to other group at same F_ap _level

^a ^p < 0.05 compared to same group at F_ap _= 0 BW

^b ^p < 0.05 compared to same group at F_ap _= 0.3 BW

**Table 3:** Micromotion results

		Group 1 No abductor		Group 2 Abductor	
**Component**	**F_ap_**	**Average**	**95% CI**	**Average**	**95% CI**

Lateral translation (μm)	0	7	± 5	2*	± 2
	0.3 BW	10 ^a^	± 4	2*	± 3
	0.6 BW	16 ^ab^	± 5	2*	± 2

Anterior translation (μm)	0	4	± 5	0	± 4
	0.3 BW	7	± 9	-4	± 16
	0.6 BW	25 ^ab^	± 8	-1*	± 19

Distal translation (μm)	0	11	± 6	9	± 3
	0.3 BW	12	± 6	9*	± 3
	0.6 BW	16 ^ab^	± 7	8*	± 2

Sagittal plane rotation (×10^-3^°)	0	0	± 1	3	± 5
	0.3 BW	5	± 5	3	± 6
	0.6 BW	11 ^ab^	± 9	1*	± 5

Frontal plane rotation (×10^-3^°)	0	14	± 6	10	± 12
	0.3 BW	20	± 6	12	± 16
	0.6 BW	35 ^ab^	± 11	10*	± 12

Transverse plane rotation (×10^-3^°)	0	-0	± 2	-1	± 2
	0.3 BW	-6	± 14	9	± 16
	0.6 BW	-61 ^ab^	± 16	14*	± 37

* p < 0.05 compared to other group at same F_ap _level

^a ^p < 0.05 compared to same group at F_ap _= 0 BW

^b ^p < 0.05 compared to same group at F_ap _= 0.3 BW

Migration occurred primarily along and about the implant axis. Distal migration accounted for 94 to 99% of the total translational migration. The average absolute rotational migration was smaller than 0.04° in the sagittal and frontal planes, but much larger in the transverse plane (rotation about the implant axis) where it reached an average of -1.2° and 0.4° for groups 1 and 2, respectively. Micromotion, on the other hand, was generally not dominated by motion in a specific direction.

Statistically, the abductor force F_abd _did not have a significant main effect on the total translational migration (p = 0.13), however, the total translational micromotion and the total rotational migration and micromotion were on average smaller with F_abd _than without F_abd _(p < 0.01), see Figures 5 and 6. In contrast, the anterior-posterior hip contact force component F_ap _had a clear significant main effect on the total translational and rotational migrations and micromotions (p < 0.01).

There was, however, a strong interaction between the abductor and the F_ap _present in all the motion resultants (p < 0.01) and all the components (p < 0.05), except the rotational migration in the frontal plane (p = 0.38). In general the abductor was only observed to affect the implant motion at F_ap _0.6 BW. With this F_ap_, all components of migration and micromotion were significantly greater without the abductor (Tables 2 and 3). The only motion components that were significantly affected by the abductor at all F_ap _levels were the rotational migration in the frontal plane, opposite in direction between the two groups, and the translational micromotion in the lateral axis, which was smaller for the abductor group.

Similarly, the effect of increasing F_ap _was mainly seen in the no abductor group. Without the abductor, increasing F_ap _from 0 to 0.3 BW increased the translational micromotion only in the lateral direction (p < 0.02). Increasing F_ap _to 0.6 BW, however, led to significantly higher micromotion in all directions (p ≤ 0.01), higher translational migration in all directions (p < 0.01), as well as higher rotational migration in the transverse plane (p < 0.01). With the abductor set-up, increasing F_ap _from 0 to 0.3 BW did not significantly affect implant motion, and increasing the F_ap _to 0.6 BW only gave a significant increase in translational migration in the lateral and distal directions (p < 0.05), and rotational migration in the sagittal plane (p < 0.01).

## Discussion

*In-vitro *mechanical tests are commonly performed to assess the effect of implant design on the stability of hip endoprostheses pre-clinically. There is no standard protocol for these tests, and the loading conditions used vary greatly. Efforts have been made to standardize the test conditions [37], however, it is not clear how the abductor muscle and the anterior-posterior hip contact force influence the translational and rotational stability of the implant. The present study examined the effect of these two parameters in the *in-vitro *assessment of cementless hip implant primary stability.

As any biomechanical investigation this study has some limitations. Composite femurs were used instead of human femurs, and the implant motion was measured at only one location. These two limitations are discussed in detail in the following paragraphs. In addition, different load magnitudes were applied in sequence to each specimen,. To minimize this effect on subsequent migration, the study was designed such that the load magnitude was applied in increasing increments simulating postoperative rehabilitation. However, during a pilot test, the micromotion observed during simulated walking was similar whether these loads were applied before or after the stair climbing cycles.

Composite femurs were used to minimize experimental variability, as was done in other studies for the same reason [13,23,38]. Their structural stiffness has been shown to approximate that of natural bone, but with less variability [39,40]. No comprehensive study comparing implant stability in composite versus cadaveric femurs was found in the literature, however, *in-vitro *tests with composite femurs [23] have yielded axial migration comparable to cadaveric femurs [41] for the CLS and press-fit Muller implants.

In our tests, the implant motion was measured at a single location. With the magnitude of physiological loads applied, the stem and the bone could not be considered rigid bodies; therefore the motion at other locations could not be determined from our experimental data. Some in-vitro studies have measured bone-implant motion at multiple locations, as reviewed by Britton et al [42], but the individual measurements are often limited to a single axis (e.g [23,43]). Experimentally, space restrictions generally translate into having to choose between measuring three-dimensional motion at limited locations and measuring uniaxial motion at several locations. With a single axis motion measurement approach, however, rotational motions between the implant and the bone can incur large errors in translational motion measurement, which are proportional to the distance between the bone-implant interface and the sensor axis. A six-degree of freedom motion measurement device enabled us to avoid such error, however, our motion measurements were limited to one location.

Two common testing set-ups were selected for this study: the first set-up applied the hip contact force alone while the second applied the hip contact force together with the abductor force. The abductor force is often included rather than other muscle groups because the abductors were demonstrated to have the most important effect of all muscle groups on stresses and strains in the proximal femur [26,28]. More complex set-ups have been used in the literature, but they are less common. For example, in one study several muscle forces (abductor, ilio-tibial band, tensor fascia latae, vastus lateralis and vastus medialis) were simulated with multiple independent actuators [10]. A set-up modeling the hip contact force alone, on the other hand, is advocated for its simplicity and reproducibility. In a previous study [13], the use of this simpler model was justified based on the reported small effect of muscles on cement stresses in cemented constructs [28].

Our measured distal migration/micromotion magnitudes for the VerSys FMT stem (walking: ~100 μm/10 μm with both set-ups; and stair climbing: 191 μm/8 μm and 385 μm/16 μm with and without the abductor force, respectively) were within the range of values reported for other cementless implants tested in composite or cadaver femurs. Distal migration/micromotion in the order of 150 μm/10 μm, 70 μm/30 μm, and 400 μm/50 μm were reported in other studies [9,10,23] for the CLS stem, a press-fit cementless implant similarly intended for proximal fixation. Stem migration measured clinically for the CLS stem, however, is substantially larger (with an average in the order of 0.7 mm at 6 months) than the reported values from in-vitro experiments [2,44]. This may be in part due to the limited number of gait cycles modeled *in-vitro *(usually 1000 or 5000 cycles) and/or the use of simpler and lower loads compared to those sometimes seen in-vivo, which may reach as high as eight times the body weight during stumbling, for example [45]. Furthermore, adaptation of the bone, i.e. remodelling and local bone resorption, may also affect post-operative implant motion. *In-vitro *tests could at best simulate resorption by milling the bone interface at a predetermined location prior to testing [46]. Nonetheless, the objective of in-vitro primary stability tests for cementless stems is not to provide an estimate of *in-vivo *migration, but to ensure that a favourable environment for successful bone ingrowth will be achieved post-operatively. It has been proposed that micromotion may be a better predictor than migration for the long-term performance of femoral implants [47], however, no clinical data was available to compare with our micromotion results.

The high torsional F_ap _loads experienced by the proximal femur during stair climbing are well documented and have been shown to occur during other activities such as jogging, fast walking, and rising from a chair [32,48,49]. Concerns have been raised that these forces may exceed the stem's torsional fixation strength [32]. However, these concerns were based on comparisons with in-vitro torsional strength assessments obtained without cranical-caudal loading on the implant [29-31], which may have underestimated the torsional strength under more physiological loading. Torsional loading has been said to affect the rotational motion of femoral hip implant [24,50]. One of these studies, however, did not apply a cranial-caudal load or measure the translational motion [49], while the other varied not only the torsional load applied, but also the muscle loads [24]. Our results indicate that for a collarless, cementless implant, increasing F_ap _not only increases the axial rotation of the implant but that the motion increases in other directions as well, particularly distally. A similar finding was reported in another study, in which stair climbing loads generated approximately 150 μm of distal migration, compared to 30 μm of proximal migration when simulating walking loads for the CLS implant [10]. In their study, however, the F_ap _(~200N, i.e. ~0.3 BW for a 70 kg individual) was smaller than the values reported for stair climbing in-vivo, i.e. 0.6 BW [32] and muscle forces also varied between their walking and stair climbing set-ups [10]. Moreover, *proximal *migration was observed under walking loads, which the authors attributed to errors inherent in their motion measurement system. The current study, on the other hand, looked at the effect of F_ap _in isolation from other parameters. Increasing F_ap _from 0 to 0.3 BW did not have a significant effect on implant motion, but a significant increase in migration (mainly in the distal direction) was observed when increasing F_ap _from 0.3 BW (walking) to 0.6 BW (stair climbing) - this effect was largest without the abductor. The micromotion also increased with increasing F_ap _(mainly in the anterior direction), but this effect was only seen without the abductor. Rotation was primarily in the transverse plane, i.e. about the implant long axis; without the abductor stair climbing produced on average 10 times higher rotational micromotion (Table 3) and 62 times higher rotational migration (Table 2) about this axis compared to walking loads. Our results therefore support our first hypothesis: the higher F_ap _loads observed during stair climbing result in greater implant-bone micromotion and migration compared with walking.

We found that inclusion of the abductor muscle force stabilized the implant both in translation and rotation, particularly when simulating stair climbing. This does not support our second hypothesis. This observation, however, is similar to another study in which inclusion of muscles (abductor, tensor fascia latae and vastus lateralis) resulted in less migration than did the hip contact force alone for a cemented implant [8]. Nonetheless, there are seemingly conflicting results in the literature; another study reported that including muscle forces (abductor, tensor fascia latae, vastus lateralis, and vastus medialis) resulted in much greater motion than did the hip contact force alone for the CLS cementless implant [10]. Although related debates[51], there is no clear explanation on this conflicting result. We suspect that these differing observations may be related to differences in medial-lateral bending moments in the femur, which are. not only affected by the abductors, but also in great part by the orientation of the hip contact force. In the study by Kassi et al. [10], the hip contact force was applied at a 20° angle from the long axis of the femur in the frontal plane, whereas in the current study and that of Britton el al. [8] it was applied at 13°. These two angles are within the range reported from in vivo measurements [15,44,52], yet they generate different bending moment distributions. At 13° from the femur axis [15], the hip contact force generates medial bending in the femur, which tapers to roughly neutral bending around the implant tip, whereas at an angle of 20° [44] it generates medial bending in the femur around the proximal stem, but substantial lateral bending at the implant tip. The abductor load generates an additional medial bending moment, which, when superposed with the effect of the hip contact force, results in a more pronounced medial moment when the hip contact force is applied at 13° compared with when the force is applied at an angle of 20°. Differences in implant-bone interface contact stresses from the resulting bending moments may explain why the muscle forces affected implant motion differently between these studies. If this is the case, the orientation of the hip contact force may be more important than whether or not the abductor force is included in *in-vitro *primary stability studies. Nonetheless, it is also possible that the effect of muscles on implant motion is sensitive to the implant design.

The muscle attachment technique may also have affected the implant motion. In one study [10] the femurs were machined at the muscle insertion site which may have artificially weakened the femur, possibly increasing in the bone-implant motion. In the current study, the abductor attachment was done through a polymethyl-methacrylate that was fitted onto the greater trochanter, and which may have reduced the motion by stiffening the bone locally. Britton et al., however, also observed a reduction in implant motion when adding muscle forces with woven polyethylene straps glued to the greater trochanter, which is unlikely to have stiffened the bone [8].

Whether it is better to include or exclude the abductor and/or other muscles during pre-clinical testing is debatable. It can reasonably be argued that including all muscles provides a more physiologically representative loading scenario. However, the question of how much bending occurs physiologically is still being argued, e.g. [53]. Inclusion of muscle forces also introduces a potential source of inter-specimen variability which could overshadow the effect of the variable being studied. Since migration measured *in-vitro *is typically lower than reported clinically, a set-up yielding higher bone-implant motion could be considered as favourable for pre-clinical testing. Based on our results, with the hip contact force applied at 13° from the femur axis in the frontal plane, maximum implant motion was observed when simulating stair climbing without the abductor force.

## Conclusions

Substantially higher rotational and translational implant motion was observed when applying an anterior-posterior hip contact force representative of stair climbing loads versus walking loads. This difference, however, was most prominent in the absence of the abductor muscle force. We believe that the current study improves upon previous research by examining the effect of the abductor force and the anterior-posterior hip contact force on implant primary stability under physiological cranial-caudal loading and in isolation from other muscle groups.

## Competing interests

The authors declare that they have no competing interests.

## Authors' contributions

YP performed the design and execution of the experimental setup and analysis, as well as drafted the manuscript. CA executed and analyzed the experiment, performed statistical analsys as well as drafted the manuscript. YY provided the design of the experimental setup, and participated in the introduction and study design. GF provided important feedback on the experimental setup and partipated in the discussion. HF provided the design of the experimental setup and participated in the discussion. TO provided important feedback on the statistical analysis and participated in the discussion.
